# Framing of visual content shown on popular social media may affect viewers’ attitudes to threatened species

**DOI:** 10.1038/s41598-021-92815-7

**Published:** 2021-06-29

**Authors:** Fernando Ballejo, Pablo Ignacio Plaza, Sergio Agustín Lambertucci

**Affiliations:** Grupo de Investigaciones en Biología de la Conservación, INIBIOMA-CONICET, Universidad Nacional del Comahue, Quintral 1250 (R8400FRF), San Carlos de Bariloche, Argentina

**Keywords:** Agroecology, Conservation biology, Human behaviour

## Abstract

Content published on social media may affect user’s attitudes toward wildlife species. We evaluated viewers’ responses to videos published on a popular social medium, focusing particularly on how the content was framed (i.e., the way an issue is conveyed to transmit a certain meaning). We analyzed videos posted on YouTube that showed vultures interacting with livestock. The videos were negatively or positively framed, and we evaluated viewers’ opinions of these birds through the comments posted. We also analyzed negatively framed videos of mammalian predators interacting with livestock, to evaluate whether comments on this content were similar to those on vultures. We found that the framing of the information influenced the tone of the comments. Videos showing farmers talking about their livestock losses were more likely to provoke negative comments than videos not including farmer testimonies. The probability of negative comments being posted on videos about vultures was higher than for mammalian predators. Finally, negatively framed videos on vultures had more views over time than positive ones. Our results call for caution in the presentation of wildlife species online, and highlight the need for regulations to prevent the spread of misinformed videos that could magnify existing human-wildlife conflicts.

## Introduction

Recent years have seen the explosive growth of social media^1–4^. These social networks offer an opportunity to produce and share content (e.g., pictures, tags, videos) that can be seen, commented on, and reproduced simultaneously by millions of people around the world. In fact, social media content may “go viral”: information may spread very rapidly, in a way that can be compared with the dynamics of infectious diseases^5^. In response to this social media growth, a new conservation field named “conservation culturomics” has emerged; it aims to use digital sources to study human–nature interactions and provide new insights into how to deal with conservation problems^6^.

Conservation culturomics offers a new opportunity to address certain aspects of the human-wildlife relationship, based on the gathering of knowledge (i.e., corpora) from web pages or video platforms. This enables the exploration of diverse issues related to conservation practices^6–8^. For instance, video platforms are an interesting data source, and can be used to evaluate what people do or think about wildlife^6^. Data shared on these platforms and other social media may provide information on cultural ecosystem services^9–11^, the preferences of tourists for observation of biodiversity, activities carried out in protected areas^12^, and the emotions these sites provoke^13^. In addition, these data can be used to evaluate illegal activities, such as hunting^7^, and to analyze people’s tolerance and perceptions of wildlife species^14,15^. Evaluating the effect of this uploaded information on viewer’s perceptions may help in planning more effective communication strategies for species conservation^16^.

Unfortunately, certain user-generated content (USG) reproduced on social media about diverse wildlife species of conservation concern (e.g., obligate scavenging birds and carnivores) could negatively influence people’s tolerance toward them^14,17^. In many cases, posted content is based on biased information without strong scientific evidence, or may even be fake news or illegal information (e.g., wildlife trading)^17,18^. This scenario can become more serious for wildlife conservation when these messages “go viral”^18,19^ or are presented with misleading “frames” (a frame refers to the way an issue is described or how a problem is conceived and approached so that it conveys a certain meaning)^20^.

People’s attitudes and decisions can be influenced by the way a message is presented^16^. Messages can be strategically framed to emphasize what really matters to the sender and the audience^20^. For example, messages framed with close psychological distance tend to be highly effective; when an object is perceived as close it tends to be perceived in more concrete way, whereas when the object is perceived as distant it tends to be construed more abstractly^21,22^. In other words, psychological distance is reduced when the framing of a message emphasizes a problem which will affect people like the viewers themselves. However, this framing could have an unintentional effect, producing cognitive bias, such as cognitive dissonance^20^. Cognitive dissonance arises when people are exposed to information that is inconsistent with their own beliefs. If this dissonance is not resolved by the audience changing their beliefs, it can lead to misperception or even rejection of the information^23,24^. In summary, the way information is presented could be a key aspect for wildlife species, since inappropriate messages may exacerbate existing human-wildlife conflict, with negative consequences for wildlife conservation.

One of the most serious human-wildlife conflicts is produced by interaction between livestock producers and carnivorous and scavenger wildlife. This conflict has been studied in many parts of the world and for different species^25–28^. Livestock producers usually argue that wildlife species such as obligate scavenging birds (hereafter, vultures) and mammalian predators are harmful, blaming them for most predation events and the associated economic loss^26,27,29^. In the case of vultures, however, scientific evidence shows that they rarely prey on livestock^25,30,31^. There is some evidence that social media could play an important negative role in this conflict, magnifying events and generating biased perceptions that may constitute an added hazard for these threatened birds^17,19^. However, the influence of social media as a potential threat (e.g., empowering existing conflicts) for vulture conservation has not yet been evaluated in depth.

Here, we evaluate the influence of social media on people´s opinions towards wildlife by analyzing viewers’ responses to content posted on a popular social medium (YouTube: http://www.youtube.com). We then propose ways to improve the presentation of information about threatened wildlife on social networks. We used vultures as a study case, as they are one of the most threatened species globally, and are currently being blamed for predation events in diverse areas of their distribution^19^. We evaluated the comments posted in response to YouTube videos showing vultures interacting with livestock. The videos were either positively or negatively framed (see details below). We also compared comments on negatively framed videos of mammalian predators interacting with livestock (e.g., felids, bears, foxes, etc.) with those on obligated scavenger vultures, to evaluate whether user perceptions vary between well-known mammalian predators and obligate scavenging birds blamed for predation events. In addition, we evaluated whether the video frame on vultures (negative–positive) could influence the number of views over time. We hypothesized that the way content was framed would influence viewer reactions, affecting their tolerance toward the species studied. We expected negatively framed videos on vultures to provoke non-empathetic reactions and negative perceptions, thus probably decreasing tolerance toward these birds. On the other hand, positively framed videos are likely to produce empathetic reactions toward vultures, and may therefore increase tolerance toward them. Moreover, although vultures rarely prey on livestock, we expected that comments with negative connotations posted in response to negatively framed videos of vultures (e.g., vultures involved in presumed predation events), and negatively framed videos of mammalian predators (e.g., bears or wolves hunting livestock) would be similar in frequency. Finally, we expected that negatively framed videos on vultures would have more views than positive ones.

## Results

**Figure 1:**
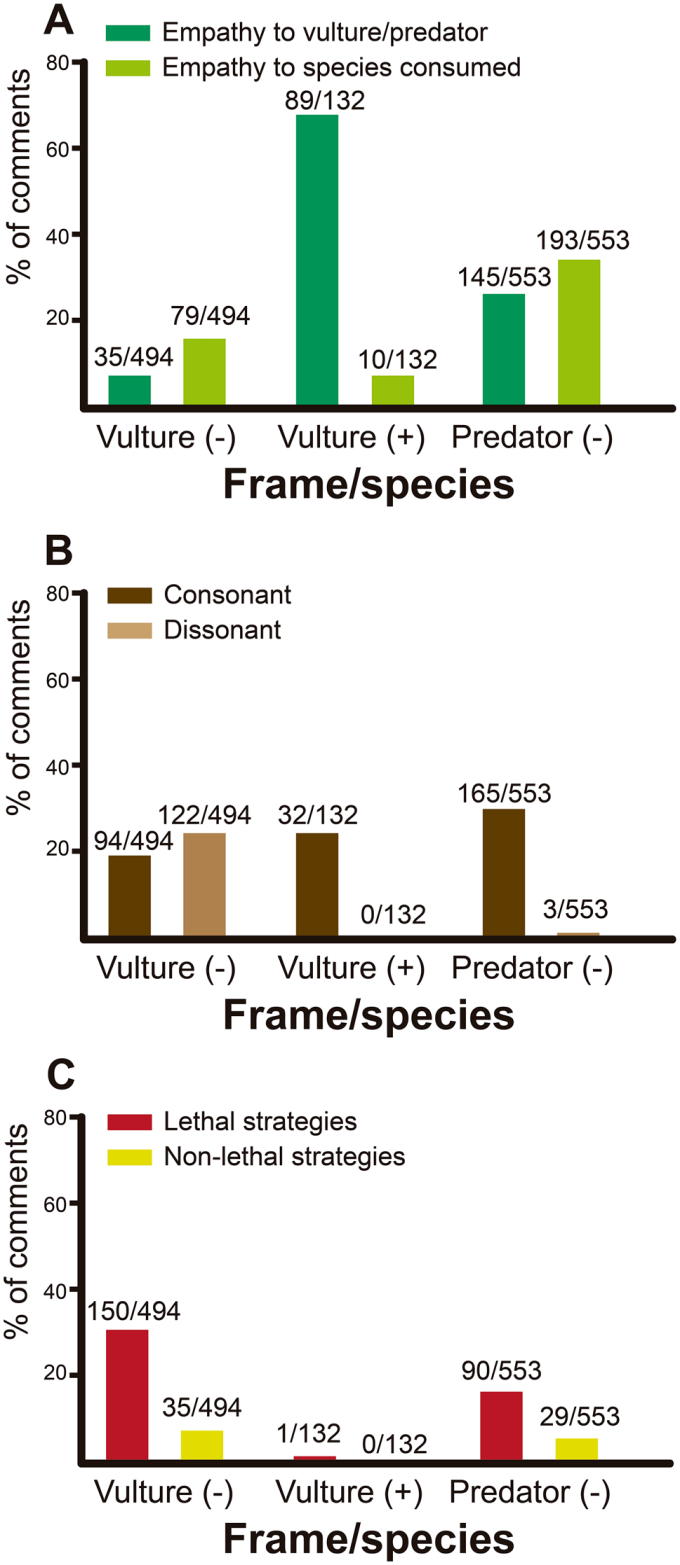
(**A**) Percentage of empathetic comments toward vultures or mammalian predators and species consumed according to video framing. (**B**) Percentage of consonant-dissonant comments in videos according to framing of video and species involved (vultures-mammalian predators). (**C**) Percentage of comments proposing lethal and non-lethal strategies for vultures or mammalian predators, according to framing of video.

Depending on the video framing (negative–positive) and the species involved (vultures-mammalian predators), the videos analyzed received different percentages of empathetic comments, consonant-dissonant comments and comments proposing lethal-nonlethal strategies to deal with the species involved (Fig. 1A–C, Supplementary Table S2). The percentage of empathetic comments posted in response to vultures tended to be higher in positively than in negatively framed videos. More empathetic comments were also posted for mammalian predators than for vultures when they were both shown in negatively framed videos (Fig. 1A). Dissonant comments were more frequently posted on negatively framed videos of vultures than positively framed videos of these birds and were also more common on negatively framed videos of vultures than negatively framed videos of mammalian predators (Fig. 1B). However, a considerable percentage of comments received (19%) were consonant with negatively framed videos of vultures where vultures were presented as predators (Fig. 1B). Finally, more comments proposing lethal strategies to deal with target species were posted on negatively framed videos of vultures and predators than positively framed videos on vultures (Fig. 1C). However, more of these comments were posted on negatively framed videos of vultures than negatively framed videos of mammalian predators (Fig. 1C).

Our models showed that message framing (negative–positive), psychological distance, and species involved (vulture-mammalian predators) all influenced the probability of receiving comments proposing a lethal strategy and empathetic comments toward the species involved. Negatively framed videos on vultures had a higher probability of receiving comments proposing a lethal strategy than positively framed videos on these birds (Estimate (± SE) = − 3.53 ± 1.17; Z-value = − 3.00; P = 0.002). Moreover, negatively framed videos on vultures had a lower probability of receiving empathetic comments toward these birds than positively framed videos (Estimate (± SE) = 3.14 ± 0.59; Z-value = 5.28; P < 0.001).

In negatively framed videos of vultures and mammalian predators, psychological distance influenced the probability of videos receiving comments proposing a lethal strategy to deal with these species. Videos with close psychological distance were more likely to generate comments proposing a lethal strategy for vultures and mammalian predators (Table 1A). Moreover, the species involved (vultures-mammalian predators) influenced the probability of generating comments showing empathy for wildlife. Videos on mammalian predators had a higher probability of generating empathetic comments than videos of vultures (Table 1B).

**Table 1:** Logistic regression mixed models to evaluate: (A) the influence of species and psychological distance on the probability of receiving comments proposing a lethal strategy to deal with species involved in negatively framed videos of vultures and mammalian predators, (B) the influence of species and psychological distance on the probability of receiving empathetic comments toward species involved in negatively framed videos of vultures and mammalian predators.

	Estimate	Std. error	z value	P
**(A)**				
Intercept	− 1.377	0.408	− 3.369	** < 0.001**
Species [mammalian predators—vultures]	0.178	0.440	0.405	0.685
Psychological distance [close-distant]	− 0.966	0.432	− 2.233	**0.025**
**(B)**				
Intercept	− 1.074	0.447	− 2.403	**0.016**
Species [mammalian predators—vultures]	− 1.795	0.499	− 3.596	** < 0.001**
Psychological distance [close-distant]	0.220	0.492	0.448	0.654

Statistically significant results are indicated in bold.

The estimates of the species and psychological distance variables correspond to “vultures” and “distant psychological distance”. The best models containing both predictors (species and psychological distance) are reported in this table (for model comparisons see Supplementary Table S3).

**Figure 2:**
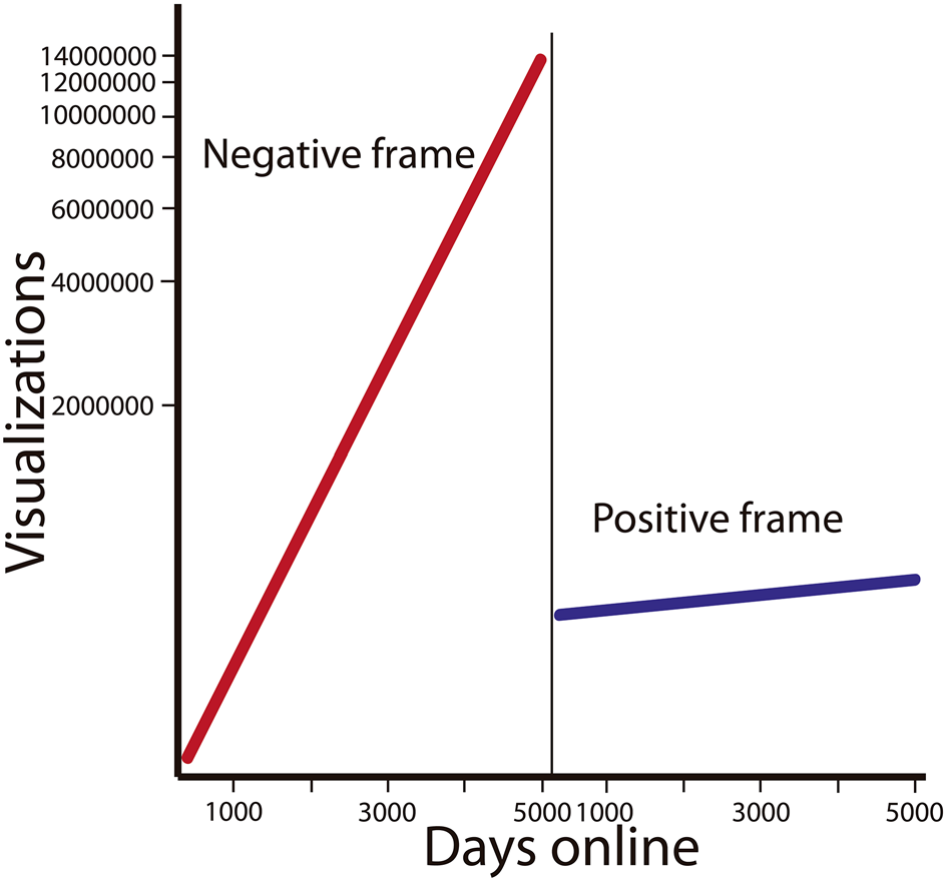
Predicted number of views of videos on vultures with negative or positive framing (see Fig. 3) in relation to the days since they were uploaded onto YouTube (based on a GLM with Poisson distribution, see “Methods”).

Finally, the interaction between video frame (negative–positive) and the number of days a video was available online influenced the number of views of vulture videos (Estimate (± SE) = − 0.0008 ± 0.0000004; Z-value = − 1693.7; P < 0.001). Videos with a negative frame had more views over time than those with a positive frame (Fig. 2).

## Discussion

**Figure 3:**
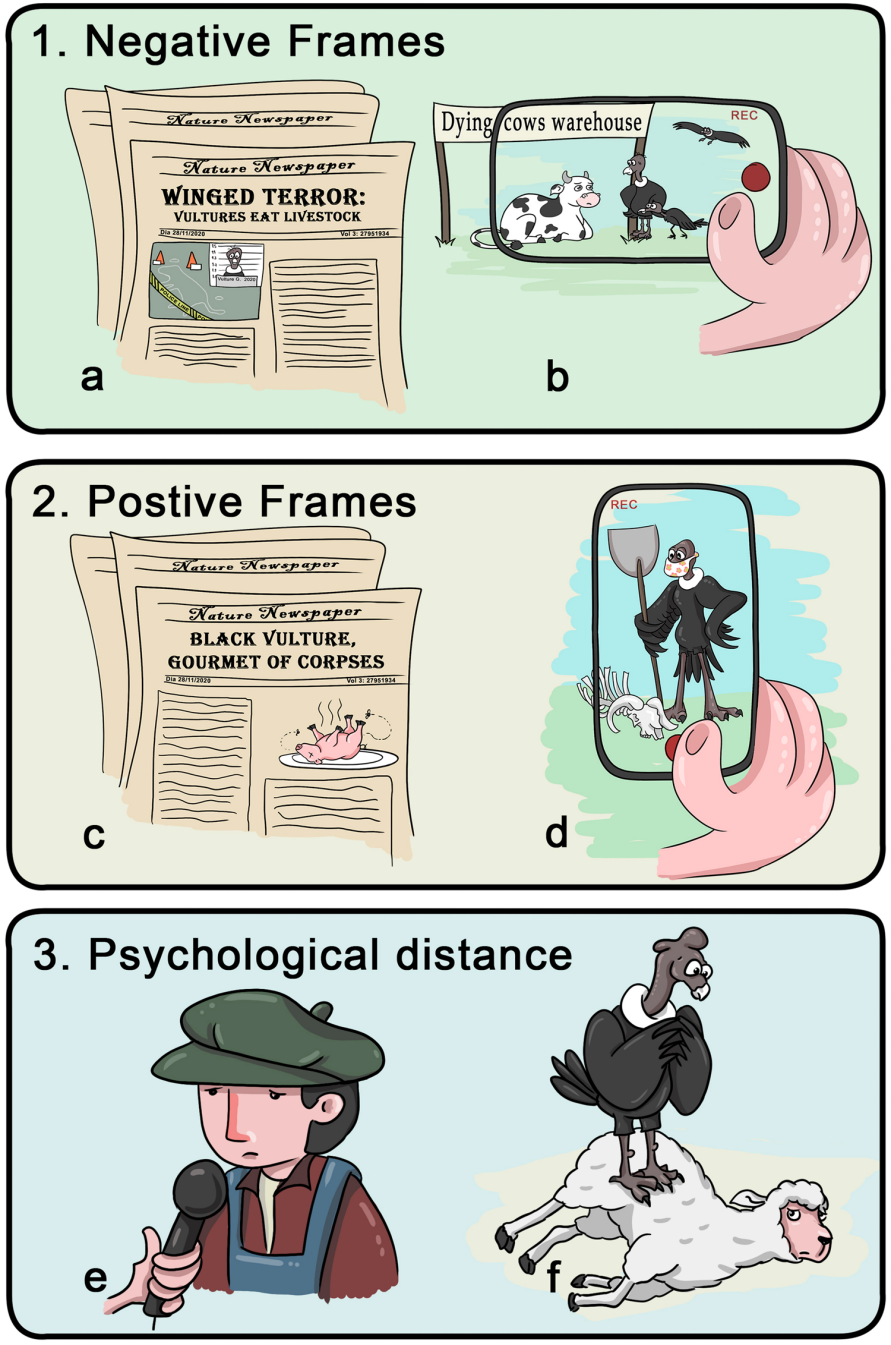
Scheme of the classification of videos according to frame. Negatively framed videos (1), including: (**a**) videos with a negative emphasis in titles or descriptions, (**b**) videos with a negative emphasis in their images. Positively framed videos (2), including (**c**) videos with a positive emphasis in titles or descriptions, (**d**) videos with a positive emphasis in their image. Videos with psychological distance frame (3), including (**e**) close psychological distance videos in which there are farmers’ testimonies about wildlife animals killing their livestock, (**f**) distant psychological distance showing wildlife interacting with livestock but no human testimonies or presence (more details in the main text).

We found that the way a video was framed influenced viewers’ opinions of the species involved, and also the number of views. For vultures in particular, the title or video description and the way the images were presented influenced the tone of the comments posted. In addition, showing testimonies of people expressing their concerns about the damage their livestock stocks may suffer (close psychological distance) were more likely to receive comments with a negative connotation (e.g., expressions of desire to kill the animal involved) than videos showing only wildlife hurting livestock, with no personal testimonies shown (distant psychological distance) (Fig. 3e). Moreover, in negatively framed videos, comments on vulture species seemed to be more negative than comments on mammalian predators. Our results contribute to the discussion on communication strategies related to biodiversity conservation^20^, and are in agreement with other culturomic conservation studies suggesting that the characteristics of content posted on social media could have an influence on the tolerance of wildlife species^14^. In addition, this culturomic approach can provide some useful tools for institutions and web platforms in terms of identification of problems and communication actions that can be taken with regard to conservation of threatened species as it does on many other social topics^32^.

It is well known that communication approaches can influence people’s attitudes, feelings and behavior^20^. Therefore, the way a message is presented is key to addressing conservation problems such as human-wildlife conflict^20^. In comparison with positively framed videos of vultures, we found that negatively framed videos of these birds had a lower percentage and probability of generating empathetic comments, but a higher percentage and probability of comments proposing a lethal strategy for dealing with them. This is of concern, considering that in most cases content posted on social media about vultures is based on anecdotic, biased information without scientific evidence to support it^17,19^. In fact, almost all the videos we analyzed that presented vultures as livestock predators showed no clear evidence that vultures were preying on livestock, and most were based only on farmers’ testimonies without showing the actual predation event (Supplementary Table S1). Importantly, some videos were later found to be fake, or misunderstandings, but were not removed from the platform or declared untrue. For instance, in a video we evaluated (number 11 in Supplementary Table S1) a farmer blames vultures for his livestock losses, even though feral dogs were actually responsible for this event (https://agroinformacion.com/el-ataque-a-un-ganadero-no-fue-culpa-de-los-buitres-sino-de-canidos-los-buitres-solo-llegaron-cuando-estaban-muertas/).

Many more dissonant comments were received by negatively framed videos on vultures than by positively framed videos on these birds, or negatively framed videos on mammalian predators (Fig. 1B). This could be because information provided in videos where vultures are hurting livestock is dissonant for people who know that vultures are obligate scavenging birds and consume mainly dead animals. Despite this, a considerable percentage of comments received (19%) were consonant with negatively framed vulture videos, in which vultures are presented as predators (Fig. 1B), suggesting that many people agree that vultures are predators rather than obligate scavenger birds. Previous studies have shown that scavenger birds may hurt livestock only on rare occasions^25,29^, but there are people who believe that these birds commonly attack and kill livestock^29,33^. In this context, a viral video showing these rare, isolated events supports or may intensify these misinformed beliefs, due to the effect of consonant cognition. In fact, this situation is observed in the tendency of Internet users to select information that is compatible with their previous beliefs (confirmation bias)^34^, and to form groups of like-minded people where points of view are polarized (echo chamber effect)^35^.

Content showing exceptional behaviors that people may consider negative could have a strong influence on their perception, as our results show (negatively framed videos have more views over time than positive ones). Furthermore, negatively framed information has a stronger effect on final perception than positively framed information^36^. Thus, a single negatively framed event about a threatened species, among others which are positively framed, will have longer lasting and more marked consequences, leading to misinformation about the species. We call for careful messaging when uploading videos on wildlife, particularly when a conflict exists and a threatened species is involved. Professional media play a primordial role and should be pioneers in this issue, reducing fake news and misinformation. The involvement of science journalists is essential, and they should be in close contact with researchers to corroborate facts and prevent the spread of misinformation.

Our results suggest that psychological distance could influence responses to content posted on social media. The videos analyzed show how viewers´ comments are more negative toward vultures and mammalian predators when they see a person who is describing the loss of their livestock or claiming damages from the authorities for their losses (close psychological distance), than when they see only the interaction between these animals and livestock, without testimonies (distant psychological distance). The media probably find that these videos attract viewers, since they cause social alarm and curiosity^17^. However, these messages may intensify conflicts that could impact negatively on threatened species such as vultures, increasing threats of action like poisoning or persecution^17,37^. In this sense, the use of close psychological distance framing may be a good strategy for the implementation of conservation measures that promote positive perception of wildlife species. For instance, videos could be shown of people who have obtained benefits from wildlife (e.g., vultures cleaning dead animals produced by livestock production) instead of showing people who have lost livestock.

Worryingly, and contrary to our predictions, empathetic comments were more likely to be posted in response to negatively framed videos about mammalian predators than negatively framed videos on vultures. Moreover, comments proposing a lethal strategy were more numerous for negatively framed videos of vultures than negatively framed videos of mammalian predators (Fig. 1C). This may be due to factors we cannot evaluate with our data, such as the fact that mammalian predators (e.g., wolves, bears or lions) are well known as potential predators of livestock, or because these species could be considered charismatic by viewers^38^. Predators such as large felines, bears and wolves could occasion livestock losses in different parts of the world^26,39–42^; for example, losses generated by pumas (*Puma concolor*) in Argentina could reach 10% of the country’s total sheep production^26^. In contrast, vultures are rarely involved in predation events: predation events by obligate and facultative scavenger birds together represent only 0.1% of the total sheep production in northwestern Argentine Patagonia^43^. Despite this evidence, viewers’ comments on vultures in response to videos uploaded onto the web generally seem to be more negative than for mammalian predators. This highlights the potential danger of showing obligate scavenging birds as predators on social media.

While using posted data on social media can be an interesting approach to the evaluation of many issues, this kind of study may be subject to bias and limitations^44,45^. For example, comments posted do not necessarily represent the thinking of all viewers; certain content may encourage individuals with a tendency to comment more often and in a particular direction^45^. While several videos showed vultures or mammalian carnivores interacting with diverse species, only a few were based on the target species or received the minimum number of comments allowing us to evaluate it. Culturomic studies are usually based on the Application Programming Interfaces (APIs) provided by platforms for analysis of the data, but these computational tools are limited for text analyses because of the complexity of the language itself; thus, ironic or referential comments, polysemous and synonymous words can lead to erroneous data interpretation^44,45^. We solved this problem by analyzing and interpreting all comments individually. Therefore, while our study could have some limitations, it constitutes a first approach to understanding how people react to content about vultures presented on social media. Our results could thus be important in promoting conservation action, taking into account the influence social media may have on tolerance of the wildlife species included in this content, particularly in the case of threatened species. Further research can evaluate this issue on other social media such as Twitter, Instagram and Facebook, to increase sample size and identify the social characteristics (e.g., nationality, gender, age) that may influence people´s perception of wildlife.

Vultures are among the most threatened avian groups in the world^46,47^, and face not only threats such as poisoning with pesticides^37,48^ and lead contamination^49,50^, but also persecution, trading of parts and food shortage^47^. This is a cause for concern given the important regulating ecosystem services that these species provide, removing organic material from the environment, which in turn could be important for public health and economy^51,52^. Most of the named threats are triggered by conflict between vultures and livestock producers^19,30,37^. Our results show that these conflicts could be exacerbated by social media, resulting in a growing problem that should be taken into account for these species. Greater caution is required in the presentation of information involving threatened species in conflict with humans, so as not to generate negative consequences.

## Conclusions and recommendations

Our results showed that the way video content is framed could influence tolerance toward wildlife species. Negatively framed content could affect the viewer’s perception, encouraging aggressive reactions towards animals, some of which may be threatened species. Most of the videos analyzed show no clear evidence of livestock being attacked by scavenger birds; however, the video frame infers that the attack really happened, which makes the video potential fake news, and may generate negative consequences for species conservation.

Social media are beginning to use more methods of control of fake news (https://www.bbc.com/news/technology-47357252). However, these control methods do not seem to be sufficiently effective, and it is necessary to extend the restrictions, without violating freedom of expression^53^. YouTube allows their users to report videos that do not comply with a series of community norms (e.g., sexual, violent or terrorism content can be denounced). Unfortunately, these norms do not contemplate the effect wildlife videos can have on threatened species. We therefore propose the incorporation of a complaint category that enables social media users to report uploaded information or frames involving wildlife species that could negatively affect their conservation.

Structural changes are also needed to prevent the exposure of individuals to fake news or misinformation. Platforms should provide consumers with some indication of the quality of the source; this could be incorporated into the algorithmic rankings of content. In addition, rather than favoring content similar to users’ previous searches, platforms could present more diverse content, thus reducing the echo chamber effect^53^ and diluting the effect of negative frames^36^. There is a need for greater care in the way messages about wildlife species are presented, especially messages about threatened species, since they could have negative consequences for their conservation. Taking regulatory action in the event of inaccurate messages about wildlife, without violating the freedom of expression, would help to improve conservation action for diverse wildlife species.

## Methods

### Sample selection

We performed searches on YouTube (http://www.youtube.com) because it is one of the most popular social networks worldwide with millions of active users^54^; it is specialized on video contents, where people can share, view, and comment all kind of videos. Moreover, this platform was used before to evaluate how social media consumption affects viewers’ tolerance toward wildlife species^e.g.^^14^. The searches were performed on the Google chrome incognito window during November 2020, filtering by relevance using the following key terms: “vultures”, “carnivores” or “predators” combined with “livestock”, “cow”, and “lamb”, both in English and Spanish. These keywords were selected because they encompass the groups of animals that are commonly involved in human-wildlife conflicts (either being attacked or blamed for attacking, producing human-wildlife conflict)^14,33^. Moreover, these keywords are used widely to search for information about human-wildlife conflict on diverse Internet platforms, social media included^55^. The aim of this search was to find videos showing obligate scavenging birds (vultures) or mammalian predators (e.g., wolves, bears, lions) from different parts of the world interacting with live or dead livestock (e.g., eating, flying around, hunting, injuring, walking around, etc.). Like other articles analyzing videos^7^, we evaluated up to 200 videos for each search we performed; these were generally the earliest videos we found related to the subject studied in this paper. We performed 18 searches combining the different keywords, resulting in 3600 videos. We excluded videos unrelated to the issue studied, videos with fewer than 10 comments that could be analyzed according to our aim, and videos not showing any species of interest or not contributing to the aims of this study. We obtained a subset of 56 videos involving vultures and mammalian predators. For each video, we recorded the following data: the title, URL, number of comments, number of views and days of permanence online since publication.

We classified each video as positively or negatively framed by analyzing the title, description and content of the videos. We focused on detecting words, sentences or images with connotations denoting the message frame, as done in other studies (e.g., Casola et al.^14^). Negatively framed videos of vultures and mammalian predators had titles or descriptions that included words such as "predation" or “killing” (Fig. 3a,b). In this category we also placed videos suggesting that vultures eat live animals (e.g., calves, lambs) instead of carrion, or videos edited to emphasize vultures injuring or intimidating livestock when still alive, in some cases without considering the entire story (e.g., not providing information on the previous events, the place, or the health status of the animal affected). Positively framed videos did not use words such as "predation" or “killing” in their titles or descriptions; they either referred to vultures eating carrion and not live animals, or were edited to emphasize the positive role of these birds in the ecosystem (e.g., cleaning carcasses) (Fig. 3c,d).

In addition, we classified videos according to their psychological distance framing, as close or distant (Fig. 3e,f)^21^. Close psychological distance videos included contents showing farmers’ testimonies about wildlife interaction with their livestock (e.g., farmers describing vultures or mammalian predators killing their livestock; Fig. 3e). Distant psychological videos showed wildlife interacting with livestock but did not include farmer testimonies (Fig. 3f). We excluded videos without a clear frame (not classifiable in the categories described above).

### Comment codification

We evaluated user-generated content by using natural language processing approaches^6,32^. In this way we extracted quantitative information about the sentiments expressed in comment posted on YouTube videos. Then, we analyzed the tone of comments based on the words (particularly the adjectives) or written expressions (e.g., kill them all with machine guns, that poor calf) posted by users (Table 2). First, one author (FB) codified the comments posted on each selected video, and this was then repeated by the other authors to verify the result; any dissidence was discussed. There were more than 95% of coincidences between authors, and the few dissidence were decided easily since at least 2 of the 3 authors supported one of the options. We evaluated up to the first 500 comments in each video, ordering them from the most recent to the oldest. Comments not related to the issue under study (e.g., political opinions, racist comments, etc.) and responses to the main comments were excluded. To standardize the data, we included only one comment from each user.

**Table 2:** Codification used for the comments posted in response to videos on vultures and predators uploaded onto YouTube.

Category	Sub-category	Definition	Examples
Empathetic comments	(a) Empathetic comments toward the species consumed or injured (b) Empathetic comments toward the vulture or mammalian predator (c) Neutral	(a) Main awareness and affection for the animal consumed or injured (b) Main awareness and affection for vulture species or mammalian predator (c) Non-classifiable in this category	(a) Poor sheep (b) Vultures are just doing their job (c) What on Earth?!
Dissonant-consonant comments	(a) Consonant: video shows the habitual behavior of a species (b) Dissonant: video shows exceptional behavior of a species (c) Neutral	(a) People who believe the video shows the habitual behavior of a species (b) People who believe the video does not show the habitual behavior of a species, or shows exceptional behavior of a species (c) Non-classifiable in this category	(a) They have done that since the planet was born! (b) These animals don’t usually hurt anything alive (c) Our government in action. Oh, brother
Strategy proposed (if available)	(a) Lethal strategies (e.g., poisoning, illegal shooting, persecution) (b) Non-lethal strategies (e.g., better livestock practices) (c) Neutral	(a) People suggesting solving the conflict using lethal strategies (b) People suggesting solving the conflict using non-lethal strategies (c) Non-classifiable in this category	(a) Kill the vultures (b) Get a dog (c) Wow, so many bird haters

Comments were grouped into the following categories: (1) empathetic comments, with the following non-exclusive subcategories: (a) toward wildlife (vulture or predators), (b) toward the livestock attacked or consumed, (c) neutral (comments non-classifiable in any previous category); (2) comments consonant with or dissonant to the opinions or images presented in the videos, with the following non-exclusive subcategories: (a) consonant comments: comments posted by people who believed that the videos were showing the natural behavior of the species involved, (b) dissonant comments: comments posted by people who believed that the video was showing uncommon or exceptional behavior for the species, (c) neutral (non-classifiable under any of the previous categories); (3) comments proposing strategies to deal with species involved (vultures-mammalian predators), with the following non-exclusive subcategories: (a) lethal strategies (e.g., poisoning or shooting), (b) non-lethal strategies (e.g., livestock guardian dogs, surveillance), (c) neutral (non-classifiable under any of the previous categories) (Table 2). We downloaded the comments of each video from the YouTube platform, and considered only videos with at least 10 comments that can be considered and analyzed under our criteria.

After all exclusion criteria had been met, we obtained a definitive sample of 25 videos (11 negatively framed on vultures, 7 positively framed on vultures, and 7 negatively framed on predators). These videos received 1179 classifiable comments (494 belonging to negative frames on vultures, 132 comments belonging to positive frames on vultures, and 553 comments belonging to negative frames on predators) (Supplementary Tables S1, S2).

### Statistical analysis

We first estimated the percentage of comments made in response to the videos analyzed, classifying them according to (i) comment category (empathetic comment, consonant-dissonant comment and strategy proposed to deal with species involved), (ii) video frame (negative–positive) and (iii) species involved (vultures-mammalian predators) (Fig. 3; Supplementary Table S2).

A set of four Generalized Linear Mixed Models (binomial distribution with Logit function) were then performed, with the video ID as a random effect. The first two models were used to evaluate a subset of videos, positively and negatively framed, showing vultures interacting with livestock. They were assessed for: (i) the influence of the predictor video framing (negative or positive) on the probability of a video receiving comments proposing a lethal strategy (e.g., shooting); (ii) the influence of the predictor video framing (negative or positive) on the probability of videos receiving empathetic comments toward vultures. We then performed two models with a subset of negatively framed videos of vultures and mammalian predators interacting with livestock, to assess the following: (i) the influence of predictor species (vulture or mammalian predators) and psychological distance (close-distant) on the probability of videos receiving comments proposing lethal strategies to deal with these species; (ii) the influence of predictor species and psychological distance on the probability of videos receiving empathetic comments toward the species involved. We performed model comparison based on AIC criteria, including both predictors (species and psychological distance) and their interactions (Supplementary Table S3). For all these models we assigned (1) to comments proposing a lethal strategy toward vultures or mammalian predators and for empathetic comments toward these species, and (0) for comments proposing non-lethal strategies toward vultures or mammalian predators and for empathetic comments toward the species consumed (e.g., sheep or cows).

Finally, a Generalized Linear Model with a Poisson distribution (Log function) was used to evaluate whether the framing of the video, number of days available online, or interactions between these two factors influenced the number of views of videos on vultures (negative and positive frame). All statistical analyses were performed with R core team (2015)^56^, and we considered P values < 0.05 as significant. The glm and lme4 packages^57^ were used to perform the Generalized Lineal Models and the Generalized Linear Mixed Models.

## Supplementary Information

Supplementary Information.
