# Supplementary material for: Framing of visual content shown on popular social media may affect viewers’ attitudes to threatened species
Source: Sci Rep. 2021 Jun 29;11:13512. doi: 10.1038/s41598-021-92815-7 (PMC8241864; doi:10.1038/s41598-021-92815-7)
Supplement: Supplementary file 1 — Supplementary Information. [file 41598_2021_92815_MOESM1_ESM.docx]

**Framing of visual content shown on popular social media may affect viewers’ attitudes to threatened species**

**Authors:** Fernando Ballejo, Pablo Ignacio Plaza, Sergio Agustín Lambertucci

**Supplementary Table S1:** Videos used, species involved, number of views, number of comments and frame classification.

| **Video title and URL** | **Species involved** | **Number of Views** | **Number of Comments** | **Classification** | **Day on line** |
| --- | --- | --- | --- | --- | --- |
| 1. El Cóndor  https://www.youtube.com/watch?v=4ljE5BSQoD8&t=104s | Vultures | 143827 | 124 | Negative frame | 2937 |
| 2. Cows defending baby calf  https://www.youtube.com/watch?v=TUpEHAeveHQ | Vultures | 1264915 | 407 | Negative frame | 3532 |
| 3. Wolf attack - Two wolves Attack the sheep  https://www.youtube.com/watch?v=ydJ3uf0An-w | Wolves | 490848 | 230 | Negative frame | 471 |
| 4. Wolf catching prey: Caught-on-camera wolf vs sheep encounter ends as you’d expect – TomoNews  https://www.youtube.com/watch?v=APKBnjkKyPc | Wolves | 118867 | 332 | Negative frame | 1614 |
| 5. Winged terror: Vultures eat livestock & pets alive in Kentucky  https://www.youtube.com/watch?v=bqRMXrF_MMc | Vultures | 42846 | 672 | Negative frame | 485 |
| 6. Black vultures are killing this farmer's cows and he needs a permit to fire back  https://www.youtube.com/watch?v=IUFpyMtvcno | Vultures | 5276 | 67 | Negative frame | 836 |
| 7. Brutal ataque de un grupo de buitres a un ganado vivo en Canencia  https://www.youtube.com/watch?v=H9gkALSHGbU | Vultures | 20269 | 65 | Negative frame | 362 |
| 8. Buitres hambrientos atacan y matan a los animales en Guadalajara  <https://www.youtube.com/watch?v=4TDlNvCqi9c> | Vultures | 84368 | 84 | Negative frame | 1676 |
| 9. Ataque de buitres a una vaca viva  https://www.youtube.com/watch?v=HIn1uF-l9sY | Vultures | 48117 | 36 | Negative frame | 1686 |
| 10. Ataque de buitres a una vaca en Bogajo  https://www.youtube.com/watch?v=e5G6ZdL-vGg | Vultures | 125122 | 43 | Negative frame | 1654 |
| 11. Buitres atacan a ovejas  https://www.youtube.com/watch?v=840fKkBxHEE | Vultures | 15002 | 76 | Negative frame | 498 |
| 12. Sorpresa en Pozoseco por el ataque de buitres a ovejas vivas. 21/06/2016.  <https://www.youtube.com/watch?v=fYQS2aK9mvE> | Vultures | 29493 | 25 | Negative frame | 1594 |
| 13. Cóndor andino (*Vultur Gryphus*) Cayambe  https://www.youtube.com/watch?v=8ytMFg552MI | Vultures | 10594559 | 54 | Negative frame | 2388 |
| 14. Wild African vulture birds scavage bones of dead animals - BBC wildlife  https://www.youtube.com/watch?v=zxj9YO4Qtx0 | Vultures | 395418 | 162 | Positive frame | 4534 |
| 15. Black Vulture, Gourmet of Corpses \| River Fox  https://www.youtube.com/watch?v=tuYQSnbd8Us | Vultures | 175385 | 66 | Positive frame | 1901 |
| 16. Vultures eating dead animal  https://www.youtube.com/watch?v=73P21XYZmQ8 | Vultures | 252765 | 129 | Positive frame | 1395 |
| 17. Vultures attacking - extreme animals – wildlife  https://www.youtube.com/watch?v=QUGyMQuavik | Vultures | 84112 | 32 | Positive frame | 2989 |
| 18. Vultures feasting on a carcass, India, 1968 from Louis Malle documentary  https://www.youtube.com/watch?v=gbp7or0ZXvo | Vultures | 1857980 | 109 | Positive frame | 3293 |
| 19. Carroñeros en acción!! (buitres, zorro, lobos..) - HD!  <https://www.youtube.com/watch?v=lWRdK4dpjSw> | Vultures, foxes and wolves | 1239058 | 112 | Positive frame | 1599 |
| 20. Aguila y Condor, ruta 9 norte, Magallanes, Chile  https://www.youtube.com/watch?v=b0ain_I6wVA | Vultures | 686477 | 259 | Positive frame | 714 |
| 21. Young Lions Kill Donkeys \| BBC Earth  https://www.youtube.com/watch?v=C3qUxhjtHdM | Lions | 1204550 | 1178 | Negative frame | 996 |
| 22. Hiena vs vaca \| Ataques de animales \| Amor naturaleza  https://www.youtube.com/watch?v=Qgm8I_KNcMc | Hyena | 2554976 | 521 | Negative frame | NA |
| 23. Wolves attack, kill Stevens County cattle  https://www.youtube.com/watch?v=2uIDas6gHQs | Wolves | 24496 | 70 | Negative frame | 2957 |
| 24. Rare Footage of a Grizzly Bear Attacking a Cow  https://www.youtube.com/watch?reload=9&v=Sd-4i0tKqCw | Bears | 2359205 | 2702 | Negative frame | 1548 |
| 25. Ataques de lobos en Asturias  https://www.youtube.com/watch?v=QuGF32atdN | Wolves | 200578 | 132 | Negative frame | 2960 |

**Supplementary Table S2:** Percentage of occurrence of comments according to comment codification (see Table 2), video framing and species involved.

|  | |  | **Empathetic comments** | | | **Consonant-dissonant comments** | | | **Strategy proposed** | |  |
| --- | --- | --- | --- | --- | --- | --- | --- | --- | --- | --- | --- |
| **Frame** | **Species involved** | **Number**  **of**  **comments** | **Empathetic comments**  **(vulture/**  **predator)** | **Empathetic comments**  **(species consumed)** | **Neutral** | **Consonant** | **Dissonant** | **Neutral** | **Lethal** | **Non-lethal** | **Neutral** |
| **Negative** | Vultures | 494 | 7.1%  (35/494) | 16.0%  (79/494) | 76.9%  380/494 | 19.0%  (94/494) | 24.7%  (122/494) | 56.3%  (278/494) | 30.4%  (150/494) | 7.1%  (35/494) | 62.5%  (309/494) |
| **Positive** | Vultures | 132 | 67.4%  (89/132) | 7.6%  (10/132) | 25%  (33/132) | 24.2%  (32/132) | 0%  (0/132) | 75.8%  (100/132) | 0.7%  (1/132) | 0%  (0/132) | 99.3%  (131/132) |
| **Negative** | Mammalian predators | 553 | 26.2%  (145/553) | 34.9%  (193/553) | 38.9%  (215/553) | (29.8%)  (165/553) | 0.5%  (3/553) | 69.7%  (385/553) | 16.2%  (90/553) | 5.2%  (29/553) | 78.6%  (434/553) |

**Supplementary Table S3.** Model comparisons to evaluate the influence of the predictor species, psychological distance (Psy) and their interaction in the probability of presence or lethal (A) and empathetic comments (B) in negatively framed videos of vultures and mammalian predators.

**A) Lethal**

| **Model** | **AIC** | **ΔAIC** | **BIC** | **Variable** | **Estimate** | **Std. Error** | **z value** | **P** |
| --- | --- | --- | --- | --- | --- | --- | --- | --- |
| **Psy** | **990.2** | **0** | 1005.0 | Intercept | -1.2696 | 0.3105 | -4.089 | **<0.001** |
|  |  |  |  | Psy | -0.9881 | 0.4361 | -2.266 | **0.0235** |
| **Species+Psy** | **992.0** | **1.8** | 1011.8 | Intercept | -1.3770 | 0.4087 | -3.369 | **<0.001** |
|  |  |  |  | Species | 0.1783 | 0.4401 | 0.405 | 0.685 |
|  |  |  |  | Psy | -0.9666 | 0.4329 | -2.233 | **0.0255** |
| **Species+Psy+ Species:Psy** | 994.0 | 3.8 | 1018.8 | Intercept | -1.3512 | 0.4941 | -2.735 | **0.0062** |
|  |  |  |  | Species | 0.1368 | 0.6284 | 0.218 | 0.8275 |
|  |  |  |  | Psy | -1.0131 | 0.6629 | -1.528 | 0.1264 |
|  |  |  |  | Species:Psy | 0.0817 | 0.8783 | 0.093 | 0.9258 |
| **Species** | 994.3 | 4.1 | 1009.2 | Intercept | -1.9450 | 0.3802 | -5.116 | **<0.001** |
|  |  |  |  | Species | 0.2521 | 0.5018 | 0.502 | 0.615 |

**B) Empathetic**

| **Model** | **AIC** | **ΔAIC** | **BIC** | **Variable** | **Estimate** | **Std. Error** | **z value** | **P** |
| --- | --- | --- | --- | --- | --- | --- | --- | --- |
| **Species** | **859.2** | **0** | 874.0 | Intercept | -0.9455 | 0.3449 | -2.742 | **0.006** |
|  |  |  |  | Species | -1.8154 | 0.5004 | -3.628 | **0.0002** |
| **Species+Psy** | **861.0** | **1.8** | 880.8 | Intercept | -1.0741 | 0.4471 | -2.403 | **0.0162** |
|  |  |  |  | Species | -1.7956 | 0.4993 | -3.596 | **<0.001** |
|  |  |  |  | Psy | 0.2205 | 0.4925 | 0.448 | 0.6544 |
| **Species+Psy+ Species:Psy** | 862.2 | 3 | 886.9 | Intercept | -0.8257 | 0.5182 | -1.593 | 0.1110 |
|  |  |  |  | Species | -2.2654 | 0.7301 | -3.103 | **0.0019** |
|  |  |  |  | Psy | -0.2059 | 0.6788 | -0.303 | 0.7616 |
|  |  |  |  | Species:Psy | 0.8709 | 0.9686 | 0.899 | 0.3685 |
| **Psy** | 869.6 | 10.4 | 884.5 | Intercept | -2.2131 | 0.4845 | -4.568 | **<0.001** |
|  |  |  |  | Psy | 0.4095 | 0.6544 | 0.626 | 0.532 |
